# Spinal cord monitoring using collateral network near-infrared spectroscopy during extended aortic arch surgery with a frozen elephant trunk

**DOI:** 10.1093/jscr/rjab174

**Published:** 2021-05-08

**Authors:** Konstantin von Aspern, Sophia Sgouropoulou, Piroze Davierwala, Michael A Borger, Christian D Etz

**Affiliations:** University Department for Cardiac Surgery, Leipzig Heart Center, Leipzig, Germany; Department of Anaesthesiology and Intensive Care Medicine, Leipzig Heart Center, Leipzig, Germany; University Department for Cardiac Surgery, Leipzig Heart Center, Leipzig, Germany; University Department for Cardiac Surgery, Leipzig Heart Center, Leipzig, Germany; University Department for Cardiac Surgery, Leipzig Heart Center, Leipzig, Germany

## Abstract

The true incidence of spinal cord injury associated with modern hybrid extended arch/descending aortic procedures utilizing a frozen elephant trunk (fET) remains unclear, and it is estimated with ~5–8%. Prolonged distal arrest without sufficient hypothermic protection as well as extended coverage of segmental arteries have been suggested to cause this complication, previously uncommon in open arch surgery. Recently, extensive clinical and experimental research led to the implementation of a new method of collateral network near-infrared spectroscopy (cnNIRS) to non-invasively monitor spinal cord oxygenation in the setting of extensive thoracoabdominal aortic repair. To date, limited experience with this method during arch procedures exists. Based on recent experiments regarding the optimal cnNIRS optode placement, we used this method for the first time during an fET procedure to document mid-thoracic paraspinous oxygenation levels.

## INTRODUCTION

Permanent paraplegia due to ischemic spinal cord injury (SCI) after procedures of any modality on the descending thoracic or thoracoabdominal aorta remains a devastating complication [1]. Even for procedures limited to the thoracic aorta (e.g. arch replacement with a stented hybrid prosthesis), the incidence of SCI is not negligible at ~5–8% and more [2, 3]. Maintaining adequate spinal cord perfusion and oxygenation is therefore paramount. The most widely used invasive methods for intraoperative spinal cord monitoring are motor- and somatosensory-evoked potential measurements. For these modalities, significant technical and human resources are required and are oftentimes not readily applicable during the postoperative course. In order to monitor the spinal cord prior to, during and after extensive aortic procedures, near-infrared spectroscopy has been introduced as a novel non-invasive and easy-to-use modality [4]. Based on the collateral network (CN) concept [5], near-infrared spectroscopy has been adapted for paraspinal CN assessment (cnNIRS) to indirectly monitor spinal cord oxygenation [4]. Previous experimental and clinical studies have proven its feasibility and revealed significant correlation between lumbar cnNIRS and spinal cord perfusion/oxygenation [4, 6]. Although near-infrared spectroscopy is routinely used at our institution during aortic procedures, to date only limited experience with cnNIRS for other CN regions (e.g. mid-thoracic placement) and/or for different aortic procedures (e.g. arch procedures) exists. The aim was therefore to utilize a modified cnNIRS placement protocol for the mid-thoracic region during aortic surgery involving the arch and proximal descending aorta. The presented case is in accordance with our institutional review board approved standard of care; additional individual patient consent was waived.

## CASE REPORT

**Figure 1:**
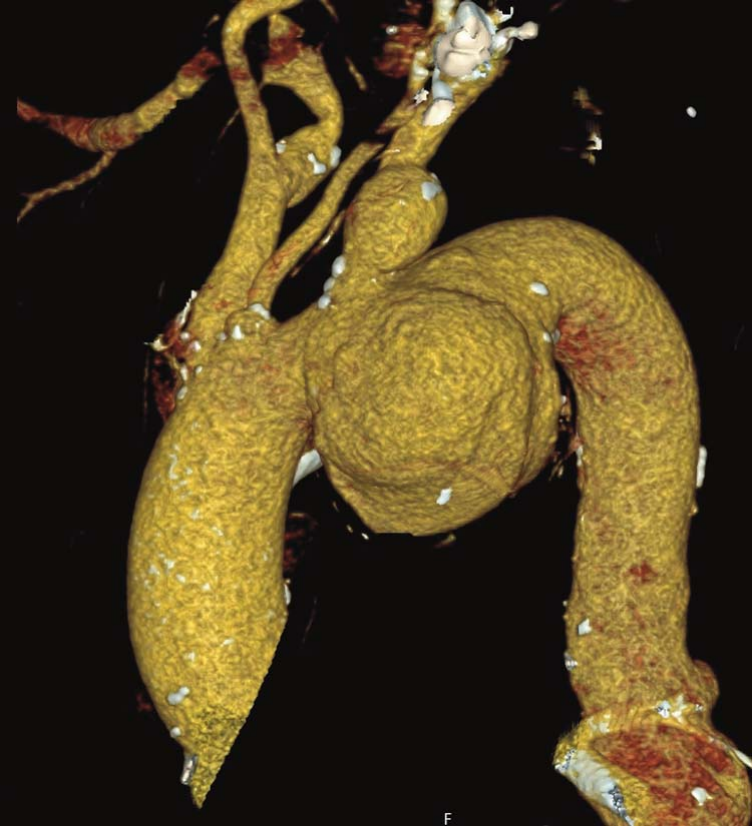
Preoperative CT reconstruction of the ascending and arch aneurysm.

A 66-year-old patient suffering from a degenerative aortic aneurysm involving the ascending aorta, the arch and the thoracoabdominal aorta was scheduled for ascending aortic and extended aortic arch replacement with a frozen elephant trunk (fET) in preparation for a second-stage thoracic endovascular aortic repair. The preoperative computed tomography scan revealed a maximum diameter of 7 cm of a berry-like sack connected to the transverse arch (Fig. 1). On echocardiography, the aortic valve was competent.

**Figure 2:**
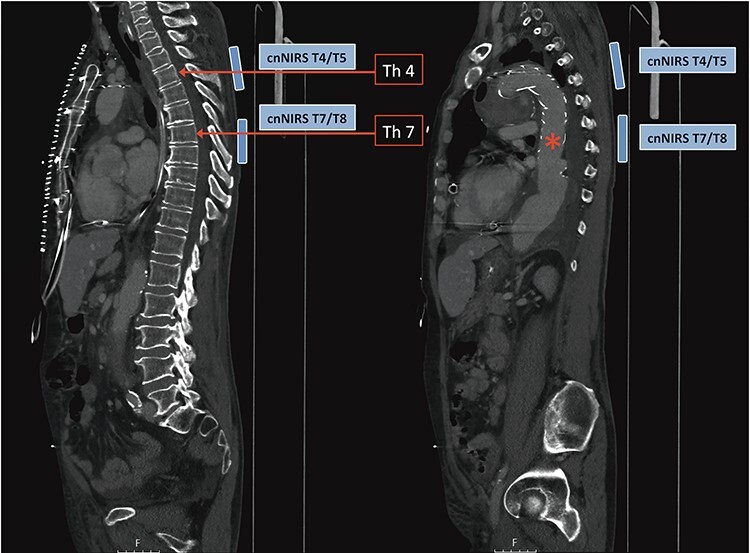
Computed tomography image demonstrating collateral network near-infrared spectroscopy (cnNIRS) optode placement for the frozen elephant trunk procedure. Optodes were placed at the high thoracic level T4/5 as reference and at mid-thoracic level T7/8 for oxygenation measurements during the procedure. (Red asterisk) Marking the distal part of the stented prosthesis.

In recent and ongoing clinical and experimental research, our group developed a translational concept for non-invasive spinal cord monitoring during and after extensive aortic procedures using cnNIRS [4, 6]. Since experimental results on the optimal cnNIRS optode placement suggested that measurements are useful from the mid-thoracic region (from T7) [7], we used this novel placement pattern for the first time during surgery. Paravertebral optodes were placed bilaterally at the level of T4/T5 for reference and at the level T7/T8 for spinal cord oxygenation estimation during the procedure (Fig. 2). In this case, cerebrospinal fluid drainage was not utilized by default prior to the procedure.

Surgery was performed through a median sternotomy with cardiopulmonary bypass (CPB) via the right axillary artery and the right atrium. According to institutional standard, bilateral antegrade selective cerebral perfusion was utilized [7]. The left subclavian artery was blocked to avoid back bleeding during arch repair. The lowest bladder temperature for circulatory arrest was 26°C. The ascending aorta and the arch were resected and a hybrid stent prosthesis was deployed prior to finalization of the distal anastomosis (Thoraflex 3036X100B). The supra-aortic vessels were anastomosed separately to the three sidearms. The intraoperative course was uneventful.

**Figure 3:**
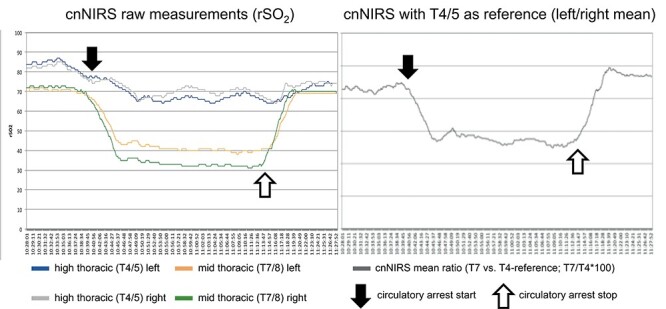
(Left) Collateral network near-infrared spectroscopy (cnNIRS) for high thoracic (T4/5) and mid-thoracic (T7/8) measurements during the procedure; (right) cnNIRS measurements as the ratio of mid-thoracic versus the high thoracic reference. (Black arrows) Start of circulatory arrest, (white arrows) perfusion start on cardiopulmonary bypass.

Baseline cnNIRS measurements for the upper- (reference) and mid-thoracic region were stable during CPB and cooling (Fig. 3, left). At the time of circulatory arrest (but with bilateral cerebral perfusion), cnNIRS measurements decreased within 10 min from 82/81 and 72/74 (100%) to a nadir of 65/67 (81%) rSO_2_ and 40/33 (50%) rSO_2_ for the upper- and mid-thoracic region, respectively. Although the reference measurements (T4/5) showed only a 19% reduction from baseline, the mid-thoracic measurements were more pronounced with a 50% reduction. When plotting the mean of the mid-thoracic cnNIRS measurements as a ratio against their respective reference (T4/5), the changes in overall oxygen saturation become more distinct (Fig. 3, right). After completion of the distal anastomosis and restoration of distal aortic perfusion (after 39 min of circulatory arrest), cnNIRS values immediately returned to baseline values over the course of 6 min (Fig. 3). The patient was extubated on day 1 following surgery and had no neurological complications during the postoperative period.

## DISCUSSION

In the presented case, cnNIRS of the mid-thoracic region demonstrated noticeable changes in the paraspinous oxygenation, which directly correlates to spinal cord oxygenation during various steps of the procedure. The aim of this report was to evaluate insights gained from ongoing research in a clinical setting as part of our translational feedback concept, where results are rapidly evaluated in a clinical setting. Recently, Kinoshita *et al*. [8] have clinically evaluated cnNIRS during arch procedures with adjunctive antegrade cerebral perfusion. Their cnNIRS setup was to position the optodes unilaterally at the high thoracic (T3) and lower thoracic (T10) CN. The authors were able to demonstrate a pronounced decrease in lower thoracic cnNIRS during the procedure, which did not recover entirely by establishing antegrade cerebral perfusion [8]. Bilateral optode placement at the mid-thoracic region during arch procedures, however, has not been evaluated to date. Similar to the aforementioned clinical study, we used the high thoracic cnNIRS position as reference. This was based on previous studies showing that oxygenation in this region remains relatively stable during aortic cross-clamping (distal to the left subclavian artery) despite of full coverage of all segmental arteries in this area [4, 6], likely due to highly sufficient immediate collateralization via the internal thoracic and intercostal arteries. In line with this finding, our recent large animal experiments have shown that changes in cnNIRS oxygen saturation in fact are evident beginning from the mid-thoracic level [9]. For open arch procedures, however, it remains uncertain whether these changes occur only as an immediate consequence of distal circulatory arrest or if insertion of the stented prosthesis also influences oxygenation measurements by cnNIRS. In case of a pronounced and unremitting decrease of cnNIRS during the fET procedure, however, alternative CPB and/or operative strategies might be considered. During circulatory arrest, neither selection of shorter fET stent grafts nor adjusting core temperatures represent viable intraprocedural options. However, initiating additional left subclavian artery perfusion (or increasing mean pressures in case selective perfusion is already established) might elevate cnNIRS values (analogous to distal perfusion in thoracoabdominal aortic repair) and in turn potentially mitigate the risk for neurologic complications. Furthermore, increasing systemic mean arterial pressures on CPB once the distal anastomosis is completed might also positively influence collateral circulation, which can be measured and potentially regulated by cnNIRS. With the evidence at hand, these considerations are only speculative and need to be further investigated. In the absence of sufficient data, future experimental and clinical studies are warranted in order to evaluate bilateral cnNIRS from the mid-thoracic region downward (T7–L5), including arch procedures with fET and different strategies in arterial perfusion.

## CONFLICT OF INTEREST STATEMENT

None declared.

## FUNDING

None.
